# P-1322. Transmission Dynamics of *Escherichia coli* Sequence Type 131 Amongst Community Dwelling Households: A Prospective Cohort Study

**DOI:** 10.1093/ofid/ofae631.1501

**Published:** 2025-01-29

**Authors:** Rebecca L Perez, Wei Cong Tan, Shweta Rajkumar Singh, Kithalaksmi Vignesvaran, Yin Mo, Aung Kyaw Thu, Rick Ong Twee Hee, Cheng Yee Tang, Ivan Seah, Ritu Banerjee, Jeanette Teo, Paul Tambyah, En Ying Tan, Wesley Yeung Lok Kin

**Affiliations:** National University of Singapore, Singapore, Singapore; National University of Singapore, Singapore, Singapore; National University of Singapore, Singapore, Singapore; National University of Singapore, Singapore, Singapore; National University Hospital, Singapore, Not Applicable, Singapore; National Centre for Food Science, Singapore Food Agency, Singapore, Not Applicable, Singapore; National University of Singapore, Singapore, Singapore; National University of Singapore, Singapore, Singapore; National University Hospital, Singapore, Not Applicable, Singapore; Vanderbilt University Medical Center, Nashville, TN; National University Hospital, Singapore, Not Applicable, Singapore; National University Hospital, Singapore, Singapore, Not Applicable, Singapore; National University Health System, Singapore, Not Applicable, Singapore; National University Heart Centre, Singapore, Singapore, Not Applicable, Singapore

## Abstract

**Background:**

*Escherichia coli* sequence type 131 (*E. coli* ST131) is a global pandemic clone associated with the rapid dissemination of CTX-M–class extended spectrum β-lactamase (ESBL) genes, conferring resistance to several antibiotic classes, and is a major cause of both healthcare and community-acquired extraintestinal pathogenic *E. coli* (ExPEC) infections. The drivers of *E. coli* ST131 pandemicity are not well-understood.

**Figure 1.**
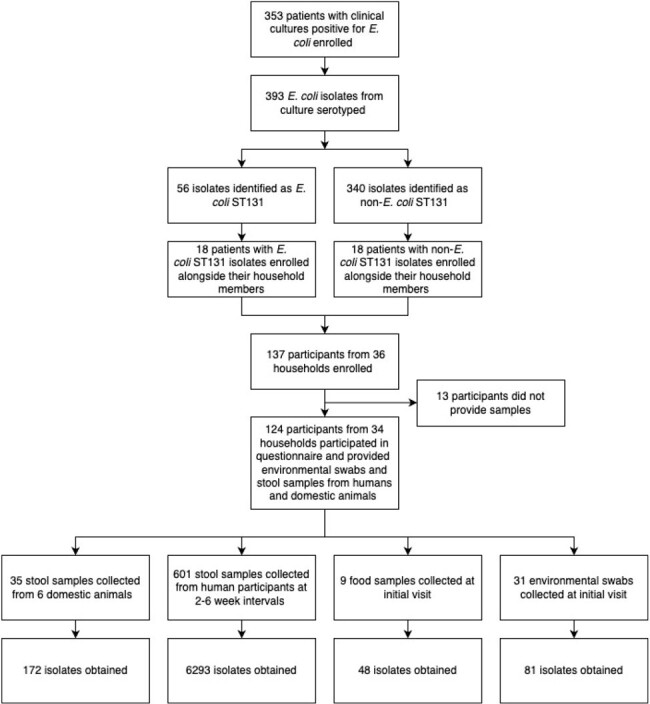
Prospective cohort study enrollment flowchart

**Methods:**

Between February 2017 to November 2018, 34 hospitalized patients with *E. coli* positive cultures (including half with ST131-positive cultures) and their household coresidents were recruited to a prospective cohort study in which all household members provided up to 12 stool samples at 2-6 week intervals. The samples were screened for *E. coli* ST131 isolates using quantitative polymerase chain reaction (qPCR), and short-read whole genome sequencing (WGS) was conducted on all *E. coli* ST131-positive isolates.

**Figure 2.**
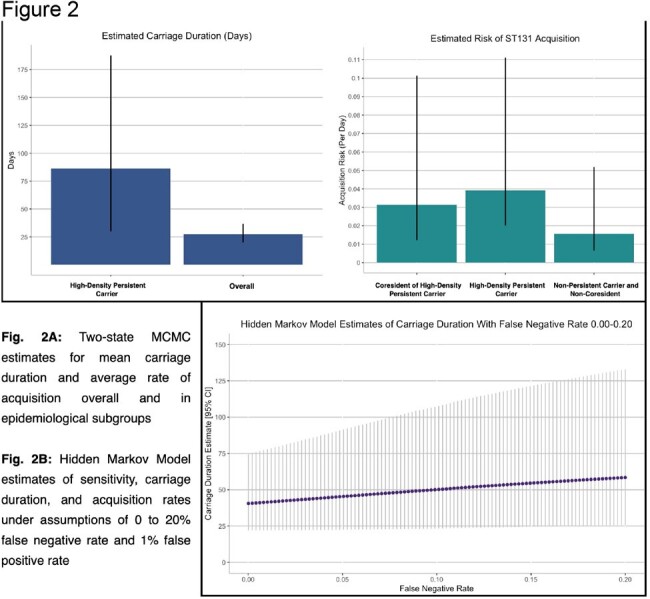
Model estimates of E. coli ST131 acquisition rate and carriage duration

**Results:**

Nine individuals (7.25%) carried *E. coli* ST131 for a median of 86.35 days [80% CrI: 30.03-188.8] (over three times higher than the cohort average of 27.33 days [80% CrI: 20.00-36.74]) and at high densities. The household coresidents of these individuals acquired *E. coli* ST131 at approximately twice the rate of those who did not coreside with a persistent high-density carrier (0.031 per day [80% CrI: .012-.10] and 0.016 per day [0.006-0.05], respectively; Odds Ratio: 2.61 [95% CI: 1.36 to 5.00] (p < 0.01)). WGS analysis identified a high degree of sequence similarity between *E. coli* ST131 isolates obtained from different individuals and at multiple non-sequential timepoints in four of the nine families in which a high-density persistent carrier was resident, indicating potential repeated transmission events.

**Figure:**
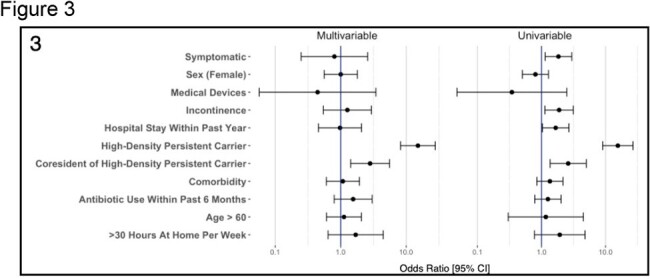

Multivariate and univariate regression analysis of risk factors associated with E. coli ST131 acquisition

**Conclusion:**

These findings indicate that a proportion of high-density asymptomatic carriers in the community may act as reservoirs for *E. coli* ST131, with frequent transmission to household contacts representing a key driver of *E. coli* ST131’s pandemic success. Further studies in other settings are needed to understand high-density persistent carrier status and address this potential community-level reservoir in order to contain this multi-resistant pathogen and reduce its clinical impact.

**Disclosures:**

**Paul Tambyah, MBBS (S'pore), Diplomate, American Board of Internal Medicine and Infectious Diseases**, Moderna: Grant/Research Support|Sanofi-Pasteur: Grant/Research Support

